# Blue-Enriched White Light Enhances Physiological Arousal But Not Behavioral Performance during Simulated Driving at Early Night

**DOI:** 10.3389/fpsyg.2017.00997

**Published:** 2017-06-22

**Authors:** Beatriz Rodríguez-Morilla, Juan A. Madrid, Enrique Molina, Angel Correa

**Affiliations:** ^1^Centro de Investigación Mente, Cerebro y Comportamiento, Universidad de GranadaGranada, Spain; ^2^Chronobiology Laboratory, Department of Physiology, Faculty of Biology, University of Murcia, Instituto Murciano de Investigacion Biosanitaria Virgen de la Arrixaca (IMIB-Arrixaca)Murcia, Spain; ^3^Ciber Fragilidad y Envejecimiento Saludable (CIBERFES)Madrid, Spain; ^4^Departamento de Psicología Experimental, Universidad de GranadaGranada, Spain

**Keywords:** lighting, alertness, psychomotor vigilance task, simulated driving, circadian rhythms, temperature, vigilance decrement, time on task

## Abstract

Vigilance usually deteriorates over prolonged driving at non-optimal times of day. Exposure to blue-enriched light has shown to enhance arousal, leading to behavioral benefits in some cognitive tasks. However, the cognitive effects of long-wavelength light have been less studied and its effects on driving performance remained to be addressed. We tested the effects of a blue-enriched white light (BWL) and a long-wavelength orange light (OL) vs. a control condition of dim light on subjective, physiological and behavioral measures at 21:45 h. Neurobehavioral tests included the Karolinska Sleepiness Scale and subjective mood scale, recording of distal-proximal temperature gradient (DPG, as index of physiological arousal), accuracy in simulated driving and reaction time in the auditory psychomotor vigilance task. The results showed that BWL decreased the DPG (reflecting enhanced arousal), while it did not improve reaction time or driving performance. Instead, blue light produced larger driving errors than OL, while performance in OL was stable along time on task. These data suggest that physiological arousal induced by light does not necessarily imply cognitive improvement. Indeed, excessive arousal might deteriorate accuracy in complex tasks requiring precision, such as driving.

## Introduction

Vigilance, or tonic alertness, is a preparatory state to optimally attend and respond to the environment (Posner and Petersen, 1990; Oken et al., 2006). Vigilance maintenance during driving is highly demanding and frequently results in both mental fatigue and sleepiness. Based on a general definition of fatigue (Williamson et al., 2011), we define mental fatigue as a biological drive for psychological restoration from prolonged or effortful cognitive activity. Sleepiness involves difficulty to stay awake due a physiological pressure to sleep, which is driven by the interaction between circadian and homeostatic factors (Borbély, 1982).

Therefore, the ability to remain optimally awake and vigilant fluctuates depending on the time of day, so that driving performance experiences the largest impairment after midnight (2 am), at early morning (6 am) and early afternoon (2 pm) (Lenné et al., 1997). Indeed, epidemiological research (Folkard, 1997; Di Milia et al., 2011) confirms that traffic accidents are most frequent at certain times, reaching their maximum around 3–5 a.m. when the levels of body temperature and vigilance are minimal. These results point out the relevance of circadian rhythms on human performance and safety.

*Circadian rhythms* are natural oscillations of biological variables with a periodicity around 24 h. The sleep-wake cycle is the most evident circadian rhythm, but other physiological processes also follow circadian variations such as hormone secretion, body temperature, arousal (non-specific physiological activation in relation to the sleep–wake axis, Oken et al., 2006) and cognitive performance, among others (Dijk et al., 1992; Bailey and Heitkemper, 2001; Schmidt et al., 2007).

The entrainment of circadian rhythms to the external day/night cycle is driven by the SCN, using the light-dark cycle as the main synchronizing cue. This process originates when photic information stimulates a class of photoreceptor cells involved in non-visual responses to light, the ipRGCs, by the excitation of their photopigment, melanopsin (Warthen and Provencio, 2012). Through the retinohypothalamic tract (Berson, 2002), this input reaches the SCN the photic signal is processed and redirected via cervical ganglia to the pineal gland, which regulates melatonin secretion. As a result, melatonin is maximally secreted in darkness, promoting sleep at night in humans, while its synthesis is suppressed in the presence of light, mediating the regulation of sleep and circadian rhythms (Cajochen et al., 2003). Consequently, exposure to light at night affects circadian rhythms and can produce SCN disruption and melatonin secretion inhibition (West et al., 2011).

In the short term, melatonin suppression at night is associated with increased arousal, which results in both difficulties for initiating sleep (Wahnschaffe et al., 2013) and changes in sleep structure (Cho et al., 2013). In the long term, chronic melatonin suppression causes chronodisruption, which has been associated with sleep disturbances (see Sack et al., 2007, for a review of circadian sleep disorders), premature aging (Ortiz-Tudela et al., 2012), metabolic disease (Garaulet and Madrid, 2010) and increased risk of cancer (Erren et al., 2010). Nevertheless, the phasic alerting effects of light may be useful to counteract the detriment derived from performing cognitive tasks at non-optimal times of day or night.

Melanopsin is maximally sensitive to short wavelength light, around 460–480 nm, i.e., in the blue color range of the light spectrum (Bailes and Lucas, 2013). Previous studies have therefore focused on the effects of blue light at night, reporting larger enhancement of activation when it was compared to mid- (green) and long-wavelength (red and yellow) lights (Cajochen et al., 2005; Cajochen, 2007; Chellappa et al., 2011).

Moreover, blue light has shown to increase arousal also during daytime, when melatonin would not be involved (Sahin and Figueiro, 2013). But according to recent findings, lights with little inhibitory activity on melatonin secretion, as short-wavelength attenuated polychromatic white light (Van de Werken et al., 2013) or long-wavelength, i.e., red color (Figueiro et al., 2009; Figueiro et al., 2015; Sahin and Figueiro, 2013), have also shown alerting effects both during daytime and night, as indexed by increased heart rate and reduced alpha and alpha-theta power of EEG. These results altogether suggest that melatonin suppression is not always necessary to increase arousal through light exposure. If so, it could be possible to improve cognitive performance through lighting, whilst avoiding the chronodisruption risks derived from melatonin suppression.

But studies testing the effectiveness of lights with different spectra over cognitive performance have yielded heterogeneous results, mostly depending on the kind of task, time of day (Gabel et al., 2015; Huiberts et al., 2015) homeostatic sleep pressure and circadian phase (Vandewalle et al., 2011; Gaggioni et al., 2014) or the previous arousal level of the participants (Correa et al., 2016), besides the light spectra itself. In addition, it is important to note that cortical, and consequently cognitive responses to light need more prolonged exposures than subcortical responses to develop, as highlighted by fMRI studies (Vandewalle et al., 2009, 2011).

Regarding our main task, driving, studies on light effects are scarce and inconclusive. While Taillard et al. (2012) found improvements of night performance under blue-light conditions, null effects have been reported as well (Phipps-Nelson et al., 2009). In the latter study, comparing blue light vs. red light and ambient (dim polychromatic light) conditions, physiological measures suggested an increase of alertness (EEG slow wave delta and theta suppression) which did not translate into behavioral benefits during driving. The effect of red light only involved a reduction of slow eye movements in comparison with DL. Different light levels between these studies (20 lx vs. approximately 1 lx, respectively) could account for their diverging results.

Given this inconsistency, we aimed to deepen into the effects of short and long wavelength light on night driving performance. We specifically compared the effects of a BWL and an OL over performance on a simulated driving task at night, employing higher intensities than the studies described above as this factor has shown to be crucial.

According to previous results, we expected light exposure to increase arousal in comparison to a control condition of DL, being this effect stronger under BWL than under the OL. In particular, under control conditions (i.e., in the DL condition) we predicted a progressive decrement of alertness along nighttime, which would be evident as: (a) an increase of the DPG, (b) higher subjective sleepiness after driving, and (c) performance deterioration along time, i.e., both increment of the position error along the driving task and slower responses in the PVT after than before driving. We expected light exposure to counteract these effects, and this lighting effect should develop progressively along time of exposure and be larger in the BWL than in the OL condition.

## Materials and Methods

### Participants

The study included thirty-six (29 women) neither-type students, according to the *Morningness - Eveningness Questionnaire* (*MEQ*) (Horne and Östberg, 1976), from the University of Granada, whose ages ranged from 18 to 25 years (mean = 21.59, *SD* = 2.54). Exclusion criteria, explored through interview, were pregnancy, major medical conditions (i.e., neurological disease, severe trauma, psychiatric history or disorders involving chronic medication), circadian or sleep disorders, night or shift work, and transmeridional travels within 3 months prior to the experiment. Participants were also asked about the use of prescribed medication during the week of the experimental session or illicit substances, which no one reported. This study was approved by the Ethics Committee of the University of Granada (n.34/CEIH/2015). All participants gave prior written informed consent and they were rewarded economically at the end of the experiment.

Participant’s chronotype and sleep quality were further checked by assessing their circadian rhythms under normal living conditions during the week prior to the experimental session.

### Materials and Procedure

#### Questionnaires and Subjective Measurements

*Morningness - Eveningness Questionnaire* (*MEQ*) (Horne and Östberg, 1976), Spanish version (Adan and Almirall, 1990): scores in this questionnaire range between 16 and 86, allowing the classification of subjects in 5 groups: *definitely evening type* (score 16–30), *moderately evening type* (31–41), *neither type* (42–58), *moderately morning type* (59–69) and *definitely morning type* (70–86).

*Karolinska Sleepiness Scale (KSS)* (Åkerstedt et al., 2014): this scale provided the subjective sleepiness level perceived by every participant, from 1 = “totally alert” to 9 = “totally sleepy, difficulties to keep on awake,” at the beginning and at the end of the experimental session.

*Mood state scale*: the participants were asked about their general mood state from 1 = “extremely negative” to 9 = “extremely positive” at the beginning and the end of the experimental session.

Visual comfort (based on the Subjective Visual Comfort assessment from Linhart, 2010) and the Rating Scale Mental Effort (Zijlstra, 1993): at the end of the experimental session, the participants evaluated their visual comfort through three different visual analog scales referring to the light they had been exposed to: “The light in this room is pleasant,” “This room is too bright,” and “I would use this kind of light for reading or working.” The participants rated their agreement with each item by placing a mark on a line ranging from “I totally disagree” (score = 0) to “I totally agree” (score = 100). Similarly, they rated their perceived mental effort during the driving task between “None” and “Extreme mental effort”.

#### Behavioral Tasks

**FIGURE 1:**
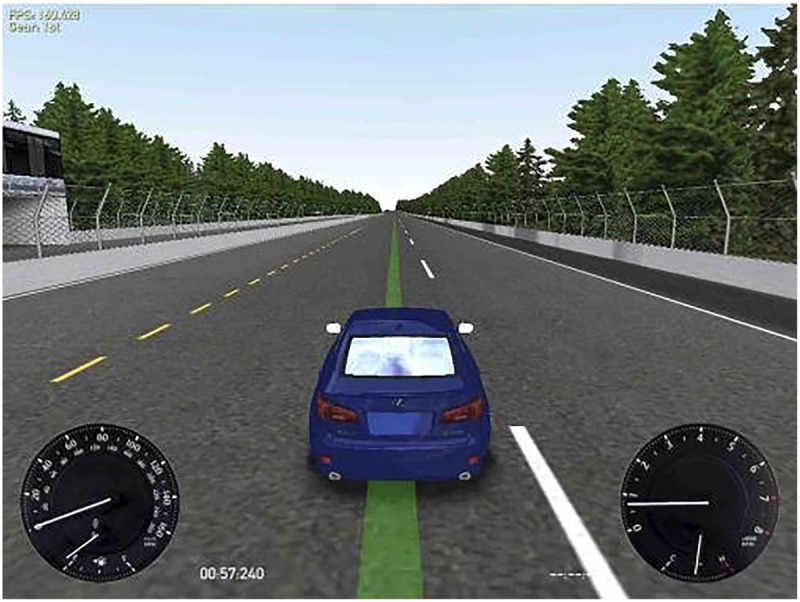
Simulated driving task display. At the left bottom corner the participants could see the velocity gauge. The participants were instructed to drive the car as centered as possible on the green line at a constant velocity of 60 miles/hour.

*Driving simulator*: the free software Racer^1^ version 0.8.9 was used to test driving performance. This system was configured by selecting the track Speedest2^2^, a road forming a big oval-shaped rectangle (approximately 3000 m long by 1750 m wide, with a bend radius of 850 m), which simulated monotonous highway driving, and thus required vigilance maintenance for long periods. The car was a Lexus IS350, which was controlled through a Logitech Momo Racing wheel and pedals set. On the display, the participants could see the car from behind and above and, at the bottom left corner of the screen, a velocity gauge (**Figure 1**). Participants were instructed to drive the car following a green line on the center of the road, keeping the car as centered on the line as possible at a constant velocity of 60 miles per hour (i.e., 96.56 km/h). As can be observed in **Figure 1**, the image contrast was high and the green line indicating the target position was clearly perceived in all conditions.

As we intended to simulate real highway driving, i.e., keeping a proper position of the car while monitoring velocity, the task was deliberately presented in the visual modality, similarly to previous research (Phipps-Nelson et al., 2009). Visual stimuli were displayed on a 24′ LCD monitor, rating 100 – 240 V∼, 50/60 Hz.

*Psychomotor Vigilance Task (PVT)*: we employed an auditory version of this computerized reaction-time (RT) task that evaluates sustained attention (Dinges and Powell, 1985), programmed through E-Prime software (Schneider et al., 2002). In the current version, the target stimulus was a 700-Hz tone of 500 ms. Participants were asked to hold their gaze at a central fixation point on the display in front of them while wearing headphones and the target was presented after a delay interval ranging randomly on each trial from 2000 to 10000 ms. The participants were instructed to respond to the target stimulus as quickly as possible by pressing a button on the same steering-wheel they used for the driving task. In every trial, the RT was recorded and displayed to the participants as feedback for 500 ms, and then the next trial began. Participants also received feedback on misses (responses after 1500 ms) and anticipations (responses before target onset). This task was presented for 10 min.

#### Physiological Measurements

A *Kronowise*^®^ambulatory circadian monitoring equipment (Chronolab, University of Murcia) was employed to assess the circadian rhythms and sleep quality of the participants during the week prior to the experimental session (see Ortiz-Tudela et al., 2010, for further information). This equipment integrates three different devices: a temperature sensor (Thermochron^®^iButton DS1921H, Dallas, Maxim) placed on the non-dominant wrist at the level of radial artery for measuring the distal temperature rhythm every 10 min; an actimeter (HOBO^®^Pendant G Acceleration Data Logger) placed on the arm for registering the rhythms of motor activity and body position every 30 s, and a luxometer (HObO Pendant Light-Temperature Data Logger) that assessed the amount of light received by the participants every 30 s along the day. Body temperature has shown to be an excellent marker of the circadian status (Kerkhof and Van Dongen, 1996; Van Someren, 2006; Sarabia et al., 2008) and, together with actimetry (Ortiz-Tudela et al., 2010, 2014) it constitutes a reliable method for ambulatory assessment of circadian rhythms and sleep. The participants were instructed to wear them 24 h per day throughout the week except for shower time. These data were analyzed by the Circadianware^®^software^3^ (University of Murcia).

Additional temperature sensors (Thermochron^®^iButton DS1921H, Dallas, Maxim) were used to assess distal (right wrist) and proximal (right clavicle) temperature every minute throughout the experimental session, as objective markers of the physiological arousal (Cajochen et al., 2005).

#### Light Manipulation

**FIGURE 2:**
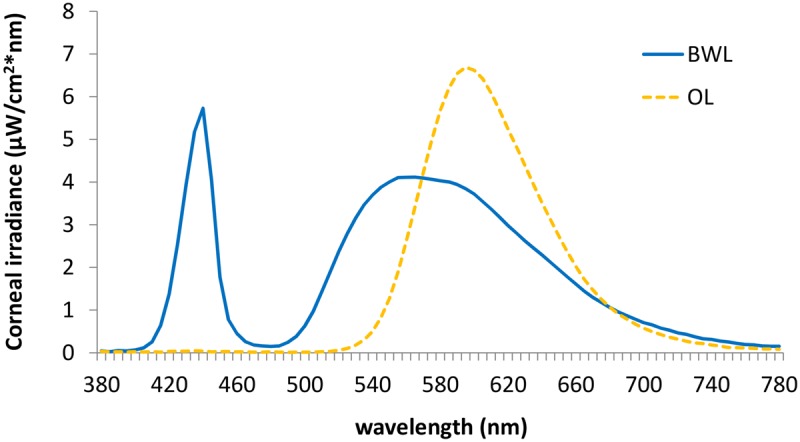
Spectral distribution of the blue-enriched white (BWL; blue line), and the orange light (OL; orange line).

We employed two 40w LED lamps, a BWL and an OL (maximum peak of spectral irradiance at 440 and 595 nm, respectively) as well as a DL control condition (lamps off in the same room, <1 lux). The lamps were placed obliquely from the left side, 60 cm far from the participants’ eyes, so indirectly falling upon both participants’ eyes and the screen, and the light emitted was diffused by a shade, mimicking a Ganzfeld full-field illumination. Illuminance at the eye level was 469 lx and 410 lx, respectively, and the spectral distribution of the lighting devices is displayed in **Figure 2**. These measures were obtained by Illuminance Spectrophotometer Konica Minolta CL-500A. All the photometric information of every lighting condition, estimated through the supplementary material from (Lucas et al., 2014) is reported in **Table 1**.

**Table 1:** Photometric values at eye level for every lighting device.

	Blue-enriched white	Orange
Irradiance (μW/cm^2^)	141.14	114.94
Photon flux (1/cm^2^/s)	4^∗^10^14^	3.54^∗^10^14^
Photopic illuminance (lux)	469	410
Cyanopic lux (α-opic lux)	323.26	4.20
Melanopic lux (α-opic lux)	224.84	26.05
Rhodopic lux (α-opic lux)	294.64	81.30
Chloropic lux (α-opic lux)	401.00	271.01
Erythropic lux (α-opic lux)	444.34	424.43


Light levels of the light emitted by the monitor were also measured with Illuminance Spectrophotometer Konica Minolta CL-500A, resulting in 0.06 lux, which was below the threshold required for causing alerting effects on the nervous system (Cajochen et al., 2000; Wood et al., 2013), and was in any case constant across the three experimental conditions.

### Study Protocol

Only volunteers whose MEQ scores ranged between 42 and 58, i.e., neither-types, were included, and randomly assigned to one of the three groups of light condition (i.e., subject 1 to DL, subject 2 to BWL, subject 3 to OL, etc.). One week before the experiment, participants were interviewed about general health and sleep habits and were informed about the study protocol. They were instructed to follow regular sleep-wake schedules during the week prior to the experimental session and were given the ambulatory circadian monitoring devices *Kronowise*^®^.

Smoke and caffeine consumption were allowed during the day of the experimental session (but not during the session itself) in line with participants’ habits, as acute withdrawal of those substances may deteriorate cognitive performance in regular consumers (Havermans et al., 2003; Rogers et al., 2005), respectively. Regular consumption of these substances was recorded, and their distribution across groups was analyzed.

**FIGURE 3:**
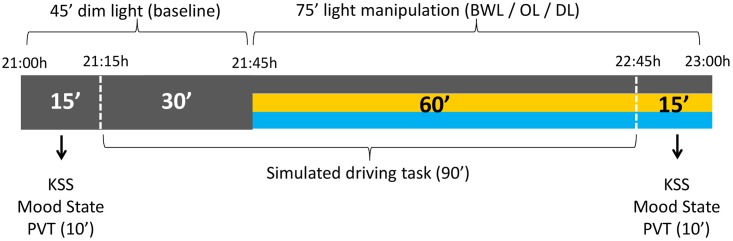
Schema of the experimental protocol. All the participants were under dim light (DL) conditions during the first 45 min, for the assessment of baseline values. The following 75 min corresponded to the light manipulation: blue-enriched white (BWL), orange (OL), or DL conditions.

The timing of light manipulation (from 21:45 h to 22:45 h) was deliberately selected to overlap with the DLMO and with the beginning of the peripheral temperature rise that follows the wake-maintenance zone and precedes sleep (Bonmatí-Carrión et al., 2014). The participants came to the laboratory at 21:00 h and stayed under DL conditions for 45 min (baseline period), during which they answered the mood state and sleepiness (KSS) scales, performed the PVT, and drove for 30 min in DL. Then, either BWL, OL, or DL was applied according to random selection and participants went on driving during 60 min. After the driving task they completed again mood and sleepiness scales and the PVT (see **Figure 3**).

### Design and Data Analysis

#### Demographic Data, Questionnaires and Subjective Measures

Mean age, mean duration of sleep the night before the experimental session (in hours), and the scores in the Cognitive Effort scale were submitted to separate one-way ANOVAs with *light condition* as between-subjects factor (BWL, OL, and DL). Analysis of the Visual Comfort scale only included two levels of *light condition* (BWL and OL).

Scores in the KSS and the Mood state scale were analyzed by a repeated-measures ANOVA with *light condition* as between-subjects factor with three levels, and *time of testing* as within-subject factor (pre vs. post driving task).

#### Circadian and Sleep Rhythms

An integrated variable “TAP” (from Temperature, Activity, and Position) was obtained from the rhythms of wrist temperature, motor activity and body position to index the general level of arousal and to infer the sleep-wake states as described in Ortiz-Tudela et al. (2010). The rhythms of motor activity, body position, wrist temperature, TAP and environmental light were then subject to non-parametric analyses (Gonçalves et al., 2015) providing several indicators referring to sleep and circadian rhythms characteristics:

**M5/L5**: five consecutive hours where skin temperature was maximal (M5) and the values of motor activity, body position and TAP were the lowest (L5). This period is identified as the main rest period.

**Midsleep time:** central time of the sleep period, located from the central time of M5/L5. It was employed as a physiological index of the sleep phase and, consequently, an objective measure of chronotype.

Finally, **sleep quality** was inferred from the L5 values of motor activity and TAP and the M5 values of skin temperature. Those were multiplied by 10, so that global sleep quality ranged from 0 (null sleep quality) to 10 (best sleep quality).

Values of sleep quality, hours of sleep per day and midsleep time averaged through the week prior to the experimental session, and hours of sleep the night prior the experimental session, were analyzed by separate one-way ANOVAs with light condition as between-groups factor.

Additionally, free-living wrist temperature during the time zone relative to our light manipulation (21:45–22:45 h) was analyzed through a 3 (light condition) × 3 (time of day: 20-min intervals from 21:45 to 22:45 h) ANOVA in order to control for possible basal differences in this trend between groups before the light manipulation.

#### Temperature Analyses

Distal (wrist) and proximal (infra-clavicular) skin temperatures were also recorded during the laboratory session as physiological indexes of the potential effects of light on the autonomic nervous system. The DPG of every participant along the experimental session was obtained by subtracting the values of the infra-clavicular temperature from the values of the wrist temperature, to minimize possible artifacts derived from analyzing the distal temperature (Kräuchi et al., 2000). Every value was baseline corrected using the last 10 min of the DL phase. These data were submitted to a 3 (light condition: blue-enriched white, orange, DL) × 3 (block) ANOVA. Further comparisons tested lighting effects on each 20-min block, as they were expected to arise after tens of minutes and evolve over time exposure (Vandewalle et al., 2007).

#### Driving Task

Analyses focused on position error, that is, the distance between the center of the car and the green line, which was rectified and corrected by velocity. The velocity error was the absolute difference between the instructed velocity (60 miles/hour) and the current velocity.

These data were analyzed as temperature data, that is, 10-min baseline corrected and submitted to a 3 (light condition: blue-enriched white, orange, DL) × 3 (block) ANOVA. Similarly to temperature analyses, the evolution of lighting effects across time of exposure was analyzed by comparing the effect of the three lighting groups on each block (baseline correction therefore minimized basal differences between subjects, therefore allowing a more strict test of light effects at block 1).

In addition to the Visual Comfort scale, we objectively checked for any potential visual effects of the light manipulation on driving performance due to either glare or other perceptual differences, which should be evident in a shorter term than non-visual effects (Vandewalle et al., 2007). Therefore, short-term variations in performance were analyzed by comparing the first 5 min of lights on with baseline (lights off), through a mixed ANOVA with *light condition* as between-subjects factor and *timing* (*pre* and *post* lights on) as within-subject factor.

#### Psychomotor Vigilance Task (PVT)

The first five trials of the task, considered as practice trials, were not included in the analysis. Data were then processed and analyzed as recommended by Basner and Dinges (2011). The RT analysis did not include responses below 100 ms (1.7% anticipations). Response speed was computed from RTs (1000/RT) to obtain a normal distribution of the data, as suggested by the non-significant Lilliefors test (*p* > 0.20) (Lilliefors, 1969). Mean response speed was submitted to a repeated-measures analysis with *light condition* (BWL, OL, and DL) as between-subjects factor and *time of testing* (*pre* and *post* driving task) as within-subject factor with two levels. Descriptive statistics and non-parametric tests were reported for the number of lapses (RTs > 500 ms), as they were very rare and showed low variability for parametric analyses. Specifically, comparisons between the number of lapses before and after driving were carried out by non-parametric Wilcoxon Matched Pairs Test, whereas between-participant differences were tested through the non-parametric Kruskal–Wallis ANOVA by ranks.

#### Bayesian Analyses

Bayesian ANOVAs were further performed to assess the probability of the null hypothesis being true. The Jeffrey-Zellner-Siow prior, and a specified effect size of 1 was selected for our analyses (Rouder et al., 2009). Hence, all null effects from the ANOVA results were accompanied by the Bayes factor, which is a ratio that contrasts the likelihood of the data fitting under the null hypothesis with the likelihood of fitting under the alternative hypothesis. Bayes factors (*B*_01_) higher than 0.33 were interpreted as support for the null hypothesis, this support being stronger with higher values. By contrast, values below 0.33 were considered as support for the alternative hypothesis, with lower values indicating stronger support (Jarosz and Wiley, 2014).

## Results

### Demographic Data and Subjective Measures

None of the groups differed in either mean age, *F*(2,33) = 1.70, *p* = 0.20, *B*_01_ = 1.739 (**Table 1**), chronotype, *F*(2,33) = 0.412, *p* = 0.666, *B*_01_ = 3.974, or perceived mental effort, *F*(2,33) = 0.783, *p* = 0.465, *B*_01_ = 3.127. The two groups exposed to light did not differ in perceived visual comfort, either in the global score or any of the items (all *F* < 1, all *B*_01_ > 0.37). Visual comfort was rated within intermediate levels (mean average: 57.83 out of 100 under blue-enriched white and 54.82 under OL).

There were no differences in the distribution of caffeine intake (χ^2^_2_ = 0.525; *p* = 0.77; 50% in blue-enriched white, 38% in orange and 36% in DL) or tobacco use (χ^2^_2_ = 1.85; *p* = 0.40; 8% in blue-enriched white, 15% in orange and 0% in DL) across groups during the day of the experimental session.

The groups were also balanced in basal sleepiness, *F*(2,33) = 1.27; *p* = 0.29, *B*_01_ = 2.246, and mood, *F*(2,33) = 0.75; *p* = 0.928, *B*_01_ = 4.996. At the end (vs. beginning) of the session, they showed higher sleepiness, *time of testing*: *F*(1,33) = 131.66, *p* < 0.01, $\eta_{p}^{2}$ = 0.8, and worse mood, *F*(1,33) = 7.76, *p* < 0.01, $\eta_{p}^{2}$ = 0.19, which did not differ between groups (*light condition* ×*time of testing on KSS scores*: *F*(2,33) = 2.2, *p* = 0.129, *B*_01_ = 2.463, and *F*(2,33) = 1.17, *p* = 0.323, *B*_01_ = 1.112, for mood scores.

### Circadian and Sleep Rhythms

In the week prior to the experimental session, mean midsleep time (BWL = 4:22, *SD* = 0:37; OL = 4:25, *SD* = 0:36; DL = 4:47, *SD* = 0:25) was typical of neither-type participants according to Roenneberg et al.’s (2003) classification, and did not differ between groups, *F*(2,33) = 1.80, *p* = 0.18, *B*_01_ = 1.642 (**Table 2**). The remaining parameters related to sleep and circadian rhythms were within the range of normality according to previous research (Ortiz-Tudela et al., 2010, 2014; Hirshkowitz et al., 2015), and well balanced between groups: sleep quality, *F*(2,33) = 0.139, *p* = 0.87, *B*_01_ = 4.369, and mean amount of sleep per night, both during the week before, *F*(2,33) = 0.821, *p* = 0.449, *B*_01_ = 2.028, and the night before the experiment, *F*(2,33) = 1.02, *p* = 0.37, *B*_01_ = 2.673 (**Table 1**).

**Table 2:** Mean scores and standard deviations (in brackets) of each group in every demographic and subjective measure.

		Blue-enriched white	Orange	Control
Age		19.5 (1.88)	20.69 (2.59)	21.46 (3.14)
MEQ score		51.83 (5.04)	49.85 (5.62)	50.82 (5.72)
Mental effort		54.17 (23.10)	58.54 (14.97)	48.36 (21.09)
Midsleep time (hh:mm)		04:22 (0:37)	04:25 (0:36)	04:47 (0:25)
Sleep hours (previous week)		7.9 (0.7)	7.5 (0.68)	7.6 (0.26)
Sleep hours (previous night)		7.4 (0.62)	7.5 (0.25)	7.1 (0.76)
Sleep quality		8.51 (0.16)	8.55 (0.29)	8.56 (0.22)
Visual comfort, global score		57.83 (22.93)	54.62 (16.71)	– –
“The light in this room is pleasant”		56.67 (23.1)	52.54 (18.68)	– –
“This room is too bright”		38.42 (27.73)	36 (21.46)	– –
“I would use this kind of light for reading or working”.		55.25 (30.02)	47.31 (30.51)	– –

KSS	Pre	3.25 (1.87)	4.31 (1.65)	3.82 (1.4)
	Post	6.67 (1.78)	6.54 (1.71)	7.09 (1.22)
Mood state	Pre	6.75 (1.49)	6.82 (0.75)	6.62 (1.5)
	Post	5.92 (1.6)	6 (1.35)	5.636 (1.75)


**FIGURE 4:**
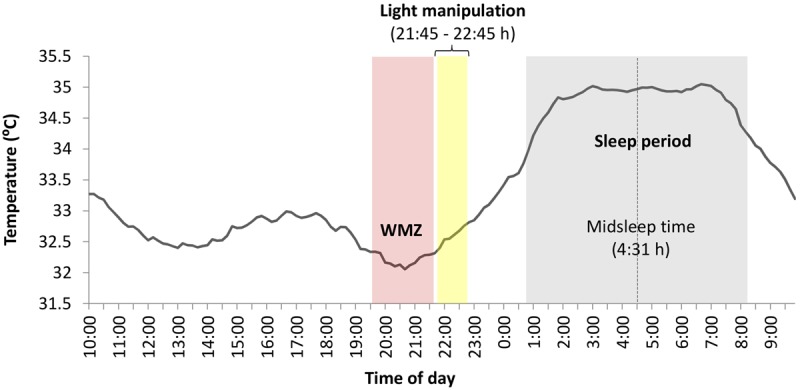
Circadian rhythm of wrist temperature averaged across participants during the week prior to the experimental session. The wake maintenance zone (WMZ, shaded in pink color), with midpoint at 20:40 h, was characterized by a dip in distal temperature values. The lighting manipulation took place an hour later, at the beginning of the distal temperature rise anticipating sleep and closely overlapping the DLMO.

**Figure 4** represents the circadian rhythm of temperature averaged across participants during the week prior to the experimental session. Inspection of **Figure 4** confirms that the light manipulation was administered after the center of the *wake maintenance zone* (nadir at 20:30 h) and matching the habitual anticipation of the sleep period. This visual impression was corroborated by the analysis of basal temperature rhythms under free-living conditions during the interval fitting the light manipulation (21:45–22:45 h). That is, wrist temperature showed the typical increase preceding sleep: *time of day F*(2,60) = 9.79, *p* < 0.01, $\eta_{p}^{2}$ = 0.25; without significant differences between groups [*light condition*: *F*(2,30) = 2.228, *p* = 0.125, *B*_01_ = 0.88; *light condition* ×*time of day*: *F*(2,60) = 2.44, *p* = 0.09, *B*_01_ = 1].

### Temperature Analysis during Driving

**FIGURE 5:**
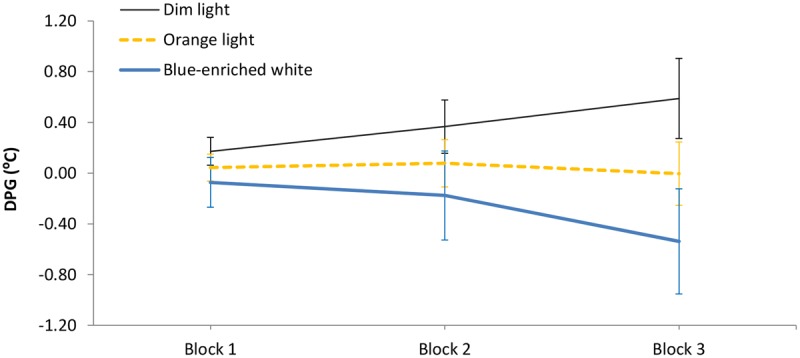
Mean Distal-Proximal Temperature Gradient (DPG) along the three 20-min blocks of light manipulation, as a function of lighting. Bars indicate standard error of the mean.

The 3 (light condition) × 3 (block) ANOVA on the DPG showed different temporal courses depending on lighting: *light condition* ×*block*, *F*(4,66) = 3.42, *p* = 0.01, $\eta_{p}^{2}$ = 0.17 (**Figure 5**). In Blocks 1 and 2, the three groups showed similar temperature gradient (all *p* > 0.11, all *B*_01_ > 2). Importantly, in Block 3, blue-enriched white decreased the DPG in relation to DL, *F*(1,33) = 6.62, *p* = 0.01, while OL did not influence temperature as compared to either DL, *F*(1,33) = 1.31, *p* = 0.26, *B*_01_ = 1.142, or blue-enriched white light, *F*(1,33) = 2.29, *p* = 0.14, *B*_01_ = 1.449). In other words, only the blue-enriched white light produced a reliable gradient decrement over time, [linear trend effect: *F*(1,33) = 5.25, *p* = 0.03].

### Driving Task

**FIGURE 6:**
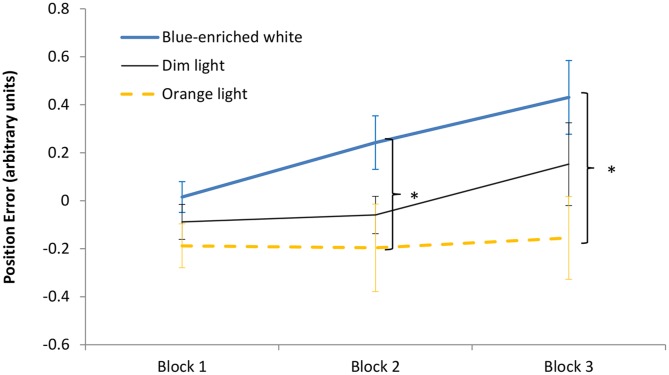
Driving task performance: evolution of the position error along the 60 min of driving task corresponding to the period of light exposure (three blocks of 20 min) in every experimental condition. Bars indicate standard error of the mean. Asterisk symbols indicate statistically significant differences.

The 3 (light condition) × 3 (block) ANOVA on the position error showed a significant effect of *block*, *F*(2,66) = 6.36, *p* < 0.01, $\eta_{p}^{2}$ = 0.16, leading to larger error with time on task. The effect of *light condition* was marginally significant, *F*(2,33) = 3.08, *p* = 0.06, $\eta_{p}^{2}$ = 0.16, but the Bayesian ANOVA rather supported the null hypothesis, *B*_01_ = 0.169. *Light condition* did not interact with block, *F*(4,66) = 1.4, *p* = 0.24, *B*_01_ = 0.578 (**Figure 6**). In Block 1, as expected, the three groups showed similar performance, *F*(1,33) *<* 1, *B*_01_ = 1.15. Interestingly, in Block 2, BWL led to larger position error than OL, *F*(1,33) = 4.79, *p* = 0.04, while BWL vs. DL showed marginal differences, *F*(1,33) = 3.04; *p* = 0.09, as supported by Bayesian ANOVA (*B*_01_ = 0.30). This lighting effect remained significant in Block 3 for BWL vs. OL, *F*(1,33) = 5.64, *p* = 0.03 [BWL vs. DL: *F*(1,33) = 1.796, *p* = 0.19, *B*_01_ = 1.337; OL vs. DL: *F*(1,33) = 0.914, *p* = 0.346, *B*_01_ = 1.962]. In other words, only the BWL group led to larger position error over time of light exposure [linear trend: *F*(1,33) = 9.58, *p* = 0.004].

The 3 (light condition) × 2 (timing: *pre* and *post* lights on) analysis of possible visual effects on performance derived from our light manipulation (e.g., glare), did not show any significant effect: [*light condition: F*(2,33) = 0.221, *p* = 0.803, *B*_01_ = 2.301; *timing: F*(2,33) = 0.001, *p* = 0.978*, B*_01_ = 4.124; interaction: *F*(2,33) = 0.782, *p* = 0.466, *B*_01_ = 30.01].

### Psychomotor Vigilance Task (PVT)

The 3 (light condition) × 2 (time of testing) ANOVA on the mean response speed showed a significant main effect of *time of testing,* reflecting slower responses after the driving task (mean response speed = 3.57; for the sake of clarity, mean RTs are reported here instead of response speed: mean RT = 280 ms; *SD* = 45) than before (mean RT = 252 ms; *SD* = 29), *F*(1,33) = 41.37, *p* < 0.01, $\eta_{p}^{2}$ = 0.56.

There was also an effect of *light condition: F*(2,33) = 3.94, *p* = 0.03, $\eta_{p}^{2}$ = 0.19 with slower responses in the BWL group (*M* = 280 ms; *SD* = 44), followed by the OL group (*M* = 270 ms; *SD* = 21) and the fastest overall RTs in the DL group (*M* = 247 ms; *SD* = 27). However, further analyses suggested that this difference was not caused by the light manipulation, since the *light condition* ×*time of testing* interaction was not significant, *F*(2,33) = 0.203, *p* = 0.818. Instead, the main effect of *light condition* might came from basal differences before driving [*light condition,* pre: *F*(2, 33) = 4.99; *p* = 0.01; $\eta_{p}^{2}$ = 0.23].

Bayesian ANOVA further supported the null hypothesis (i.e., no effect of light), regarding both the main effect of *light condition* (*H*_01_ = 1.986) and the *light condition* ×*time of testing* interaction (*H*_01_ = 0.519).

Descriptive statistics indicated higher number of lapses in the PVT after (*M* = 0.78, *SD* = 1.89) than before (*M* = 0.08, *SD* = 0.34) the driving task, which was significant according to the Wilcoxon test, *T* = 14.5, *Z* = 2.766, *p* = 0.006. On the other hand, lapses did not differ between groups, either before, *H*_2_ = 1.916; *p* = 0.384, or after driving: *H*_2_ = 1.171; *p* = 0.338.

## Discussion

It is known that driving performance undergoes detriments at non-optimal times of day (Correa et al., 2014). Since exposure to light enhances alertness (reviewed by Cajochen, 2007), our study explored the influence of light on prolonged simulated driving at night. We specifically compared, with respect to a control condition of DL, the effects of two lights with very different ability to stimulate melanopsin ganglion cells: a short-wavelength light (blue-enriched white color) expected to both enhance alertness and inhibit melatonin secretion (Lewy et al., 1980), and a long-wavelength light (orange color), assumed to moderately increase alertness without melatonin suppression (Figueiro et al., 2009, 2015; Sahin and Figueiro, 2013). We expected both lights to improve driving performance along time on task, the effect being larger for the short wavelength.

Lighting effects were further assessed by subjective and objective measurements (the KSS and the psychomotor vigilance task, respectively), including skin temperature as a physiological index of both the circadian state and phasic changes in arousal (Cajochen et al., 2005). These measures revealed a clear dissociation between the physiological and cognitive responses to light, as highlighted by neuroimaging studies (Vandewalle et al., 2009) and supported by Huiberts et al. (2016). At the physiological level, our temperature analyses showed an effect of lighting. Wrist temperature, recorded in normal living conditions, consistently showed the typical circadian rise from 21:45 to 22:45 h in all groups during the week prior to the experimental session, suggesting an arousal decrement in anticipation of sleep at night. Most relevant, during the experimental session, BWL led to a significant decrement of the DPG along time of exposure. This effect confirmed the effectiveness of our lighting manipulation by producing a reliable alerting effect at the physiological level, in line with previous literature (Cajochen et al., 2005), starting after 20 min of lighting and developing progressively along time. This effect was not found in DL (control) or in the OL condition.

Interestingly, this physiological arousal was neither reflected in our subjective assessments nor translated into better performance in the cognitive tasks: all the groups showed significantly higher somnolence in the KSS, worse mood state and slower responses in the PVT after the driving task, regardless of the lighting condition. The PVT is sensitive to vigilance fluctuations under non-optimal circumstances (Van Dongen and Dinges, 2005; Tucker et al., 2009) and has been modulated by blue light in previous studies (Phipps-Nelson et al., 2009; Chellappa et al., 2011). However, several studies from independent laboratories have also reported null effects both under blue-enriched (Gabel et al., 2015; Correa et al., 2016; Huiberts et al., 2016; Borragán et al., 2017) and red light (Figueiro et al., 2009). Bayesian analyses on our PVT data strengthened the conclusion that lighting effects on behavioral performance are not, by necessity, a standard finding in the literature (see also Borragán et al., 2017).

Most important to our study, in the driving task, the group exposed to blue-enriched white light revealed an increment of the position error after 20 min of lighting. This effect could be due to an increase of arousal, as shown by the temperature analysis, which could overtake the optimal level required for proper performance in this task (Yerkes and Dodson, 1908). According to the Yerkes–Dodson law, the level of arousal required for optimal performance depends on task difficulty: while more complex or precision-requiring tasks such as our driving task would require lower levels of arousal to facilitate concentration, simpler tasks such as the PVT would require higher levels of arousal to increase motivation. This theory holds that, in difficult tasks, the higher arousal level up to an optimum point, the better performance; beyond this critical point, any additional increment of arousal would result in performance detriments. More moderate alerting effects might be more beneficial for cognitive performance in this situation. Thus, it is important to emphasize that exposure to blue light should not necessarily improve performance; moreover, it could even impair it (Huiberts et al., 2015). However, this intriguing finding should be replicated in future studies (note that the light × block interaction was not significant, and that the clearest differences concerned the blue vs. OL contrasts).

Regarding light and driving performance, there are still heterogeneous results in the scarce existing literature. For example, in the study by Phipps-Nelson et al. (2009) blue light at night did not improve driving performance in comparison to red and polychromatic white light conditions, even though the physiological measurements (suppression of EEG slow wave delta and theta activity) suggested increase of arousal, as our temperature did. In contrast, Taillard et al. (2012) found improvements of night driving under blue-light conditions comparable to the effects of caffeine. There are relevant methodological discrepancies between these studies and ours: although both involved more adverse conditions (more prolonged driving at later times of night), Phipps-Nelson et al. (2009) employed DL intensities (1.12–1.18 lux, 2 mW/cm^2^), which could be below the threshold required to affect complex cognitive processes, while Taillard et al. (2012) used low-moderate intensities (20 lux, 7.4 mW/cm^2^). Following Yerkes and Dodson’s law, different lighting intensities are likely to bring different alerting effects (Cajochen et al., 2000) and, consequently, different cognitive responses in similar tasks. But it is important to note that our study is not comparable to those because of the considerable differences in the time of testing and duration: 2-h sessions at 21:00, 24:00, 03:00, and 06:00 h in the study of Phipps-Nelson et al. (2009) and at 1:00 and 3:15 h in Taillard et al. (2012).

The possibility that our results could be due to differences between groups was further considered. Analyses of basal measures before the lighting manipulation only showed a statistically significant difference between groups, regarding response speed in the PVT. This finding fits with our previous research reporting high individual differences in basal vigilance as measured by the PVT (Correa et al., 2016). In the current study, the null finding of light × timing interaction suggested that this basal difference did not evolve differently as a function of light. In any case, potential differences between groups were minimized by baseline correction, and supported by the finding of null effects of light on both performance and temperature during at least the first 20 min of exposure. In this line, possible differences in the rhythms of free living temperatures at the time of our experimental manipulation were also carefully considered and discarded.

It might be argued that the current behavioral results were influenced by the effects of light on visual perception, for example, by impaired performance because of a glare effect or visual fatigue. If so, glare effects should occur in both BWL and OL conditions; however, there were differences in performance between these two light conditions. Moreover, visual effects should be noticeable right at the beginning of the light manipulation phase, but no differences between conditions were found in the first minutes of the task. A differential effect of glare between groups was also unlikely on the basis of subjective assessments of visual comfort: participants’ ratings were similar between BWL and OL conditions regarding both room brightness (38/100 and 36/100, respectively) and pleasantness (57/100 and 53/100).

In any case, we can conclude that exposure to BWL in the early night does not necessarily lead to better cognitive performance, at least not under any condition increasing arousal (Correa et al., 2016). As a conclusion, our results supported a dissociation between physiological and cognitive responses to light, as previously highlighted (Vandewalle et al., 2009). Moreover, given similar physiological effects, the cognitive responses might broadly vary depending on the complexity of the task (e.g., Gabel et al., 2015). This factor should therefore be considered when interpreting the results of studies including different measurements.

Such dissociation brings up relevant implications, such as careful consideration to the popular claim that light exposure is a remedy for improving cognitive performance under any circumstances (see also Veitch, 1997). Further, facing this dissociation and considering the costs of melatonin suppression, the unconditional use of blue light at night could be no longer justified in order to improve performance. Additional research is necessary to determine which kind of cognitive processes, under which circumstances, may actually improve due to lighting stimulation at night.

## Ethics Statement

This study was carried out in accordance with the recommendations of the Ethics Committee of the University of Granada with written informed consent from all subjects. All subjects gave written informed consent in accordance with the Declaration of Helsinki. The protocol was approved by the Ethics Committee of the University of Granada (n.34/CEIH/2015).

## Author Contributions

Conceptualization: AC and JM. Data curation: BR-M, JM, EM, and AC. Formal analysis: BR-M, JM, EM, and AC. Funding acquisition: AC and JM. Investigation: BR-M, JM, EM, and AC. Methodology: BR-M, JM, EM, and AC. Project administration: AC and JM. Resources: JM and AC. Software: BR-M and EM. Supervision: JM and AC. Validation: BR-M, JM, EM, and AC. Visualization: BR-M, JM, EM, and AC. Writing – original draft: BR-M. Writing – review and editing: JM and AC.

## Conflict of Interest Statement

The authors declare that the research was conducted in the absence of any commercial or financial relationships that could be construed as a potential conflict of interest.

## Footnotes

1 http://www.racer.Nl/

2 http://www.racer-xtreme.com/

3 https://kronowizard.um.es/

## Abbreviation

BWL: blue-enriched white light
DL: dim light
DLMO: dim light melatonin onset
DPG: distal-proximal temperature gradient
ipRGCs: intrinsically photosensitive retinal ganglion cells
KSS: Karolinska Sleepiness Scale
MEQ: Morningness - Eveningness Questionnaire
OL: orange light
PVT: psychomotor vigilance task
SCN: suprachiasmatic nucleus
